# Long noncoding RNAs (CTC-471J1.2, *NeST*) as epigenetic risk factors of active juvenile lupus nephritis: a case-control study

**DOI:** 10.1186/s12969-023-00945-1

**Published:** 2024-04-27

**Authors:** Mohamed M. Zedan, Ali Sobh, Alshimaa Magdy, Mai S. Korkor, Zeinab R. Attia, Nada Khaled, Yousra Sadeq, Ahmed Hazem El-Nagdy, Ahmed E. Taha, Mohamed Ahmed Noureldin, Mohamed Taman, Doaa Mosad Mosa, Marwa H. Elnagdy

**Affiliations:** 1 Department of Pediatrics, Mansoura University Children’s Hospital, Mansoura University Faculty of Medicine, Mansoura, Egypt; 2 Department of Medical Biochemistry and Molecular Biology, Mansoura University Faculty of Medicine, Mansoura, Egypt; 3 Mansoura University Children’s Hospital, Mansoura University, Mansoura, Egypt; 4 Department of Clinical Pathology, Mansoura University Faculty of Medicine, Mansoura, Egypt; 5 Department of Microbiology, Faculty of Dentistry, Horus University, Damietta El Gadeeda, Egypt; 6 Medical Microbiology and Immunology Department, Faculty of Medicine, Mansoura University, Mansoura, Egypt; 7 Microbiology and Immunology Unit, Department of Pathology, College of Medicine, Jouf University, Sakaka, 72388 Saudi Arabia; 8 Department of Pediatrics, Horus University Faculty of Medicine, Damietta, Egypt; 9 Department of Obstetrics and Gynecology, Mansoura University Hospital, Mansoura Faculty of Medicine, Mansoura, Egypt; 10 Department of Rheumatology& Rehabilitation, Mansoura University Hospitals, Mansoura University Faculty of Medicine, 60 Elgomhoria St, Mansoura, 35516 Egypt; 11 Department of Basic Medical Sciences, Faculty of Medicine, New Mansoura University, Mansoura, Egypt

**Keywords:** Long-non-coding RNA, CTC-471J1.2, *NeST*, Lupus nephritis

## Abstract

**Background:**

Measurement of the circulating levels of long-non-coding RNAs (lncRNAs) in lupus nephritis (LN) patients could dramatically explore more insights about the disease pathogenesis. Hence, we aimed to quantify the level of expression of CTC-471J1.2 and *NeST* in LN patients and to correlate it with the disease activity.

**Method:**

This case-control study was conducted on a group of children with juvenile LN attending to Mansoura University Children’s Hospital (MUCH). Demographics, clinical, and laboratory findings were collected besides the measurement of lncRNAs by quantitative real-time PCR.

**Results:**

The expression level of lncRNAs-CTC-471J1.2 was significantly down-regulated in children with active LN versus inactive cases or controls. In contrast, the *NeST* was significantly up-regulated in active LN cases. A significant correlation was found between CTC-471J1.2 expression and LN activity parameters. Additionally, both lncRNAs showed a reasonable sensitivity and specificity in differentiation of active LN. A regression analysis model revealed that CTC-471J1.2 and *NeST* were independent predictors of active nephritis.

**Conclusion:**

The expression level of circulatory lncRNAs-CTC-471J1.2 and *NeST* can be used as sensitive and specific biomarkers for active LN. Furthermore, both could serve as predictors for nephritis activity.

## Introduction

Juvenile systemic lupus erythematosus (JSLE) is a systemic heterogeneous autoimmune disease characterized by various clinical manifestations involving different tissues [1]. Lupus nephritis (LN), which is considered one of the most severe manifestations of SLE, affects up to 70% of children with a significant impact on the disease outcome [2]. SLE complex pathogenesis has not been fully elucidated, there is a possible association of SLE with environmental factors, genetic, epigenetic expression, innate, and adaptive immunity ends with immune complexes deposition in the tissues [3, 4].

Conventional immunological serum biomarkers cannot offer specificity and/or sensitivity for full LN assessment and renal biopsy is still the gold standard to confirm the diagnosis, disease staging, and severity assessment [5].

More than 80% of the human genome is transcribed into RNA transcripts without protein-coding potential. These long-non-coding RNAs (lncRNAs) are long RNA segments longer than 200 nucleotides. They are located in either the nucleus or cytoplasm. They can affect gene expression by different mechanisms through interactions with transcription factors or epigenetic modifiers [6]. Measurement of plasma or serum levels of lncRNAs makes them potential and non-invasive biomarkers for the assessment of disease activity and prognosis [7].

There are different lncRNAs that can be used as a new hotspot in SLE. They are accurate biomarkers with high throughput, some of them can be beneficial in diagnosis, and others could serve as next-generation biomarkers to differentiate SLE patients with LN from those without [8].

CTC-471J1.2 is an example of lncRNAs located on chromosome 19. It has been shown to exhibit high sensitivity and specificity as a diagnostic marker for LN. Expression of CTC-471J1.2 has been shown to display a negative correlation with disease activity scores in all SLE patients and a positive correlation with estimated glomerular filtration rate (eGFR) only in LN patients [9].

The lncRNA Nettoie Salmonella pas Theiler’s (*NeST)* formally known as Theiler’s murine encephalomyelitis virus persistence candidate gene 1 (Tmevpg1) is an enhancer-like lncRNA. It is expressed in T helper 1 cells, CD8+ T cells, and natural killer cells. It is located adjacent to the interferon gamma (IFN-γ) encoding gene so, its expression leads to the enhancement of IFN-γ production and that could participate in the pathogenesis of SLE [10].

The assessment of the circulating levels of lncRNAs in juvenile LN patients could dramatically explore more insights into the disease pathogenesis. Hence, we aimed with this study to evaluate the expression of CTC-471J1.2 and *NeST* in LN patients and to evaluate whether their expression may play a role in the pathogenesis of the disease.

## Methods

### Subjects

This case-control study was conducted on 61 patients who attended to Mansoura University Children’s Hospital (MUCH), Mansoura, Egypt diagnosed with JSLE and forty age and sex-matched healthy children as controls. It was done in the period from August 2021 to October 2022. Cases were classified according to the 2019 European League Against Rheumatism/American College of Rheumatology (EULAR/ACR) classification criteria for SLE [11].

*Inclusion criteria*:

1. JSLE patients with lupus nephritis. Diagnosis of LN was defined as children with proteinuria > 0.5 g /24 h and/or proteinuria > 3+ and/or cellular casts (erythrocyte, granular, tubular, or mixed) and confirmed by renal biopsy [12].
2. Patient whose parents gave informed consent to be included in the study.

*Exclusion criteria:*

Cases with SLE without lupus nephritis, patients who had other conditions can affect the expression of these epigenetic factors as diabetes mellitus, malignancies, or those with a diagnosis of other connective tissue diseases.

### Data collection

Data were collected from our medical files and interpreted with respect to the demographic, clinical, disease assessment parameters, and laboratory features of the disease as follow:

1. Disease assessment including, JSLE disease activity and cumulative damage was measured using the SLE Disease Activity Index 2000 (SLEDAI-2 K) [13] and the Systemic Lupus International Collaborating Clinics/ACR (SLICC/ACR) Damage Index [14]. No activity (SLEDAI = 0), mild activity (SLEDAI = 1–5), moderate activity (SLEDAI = 6–10), high activity (SLEDAI > 10) [15].
2. Renal SLEDAI, which consists of the four kidney related parameters of the SLEDAI-2 K: haematuria, pyuria, proteinuria, and urinary casts, each item in the renal SLEDAI is assigned four points. Thus, scores for the renal SLEDAI can range from 0 to a maximum of 16 [13].
3. Pathological grading of LN is categorized according to the International Society of Nephrology (ISN) and Renal Pathology Society (RPS) 2003 criteria [16]. Active LN defined as LN class III/IV with National Institutes of Health (NIH) activity index ≥ 10. NIH activity index score ranges 0 – 24. This activity score is based on the histological characteristics of active inflammation: endocapillary hypercellularity with/without leukocyte infiltration, karyorrhexis (fibrinoid necrosis), rupture of the basement membrane, fibrocellular crescents, subendothelial deposits (wire loops), and intraluminal immune aggregates (hyaline thrombi). NIH chronicity index score ranges 0 –12. The chronicity index reflects the damage features: glomerular sclerosis (segmental or global), fibrous adhesions or fibrous crescents, interstitial fibrosis, and tubular atrophy [17].
4. Laboratory investigations:
  - Complete blood count (CBC), erythrocyte sedimentation rate (ESR), C-reactive protein (CRP), liver function tests, serum creatinine, complement (low C3 < 90 mg/dl, low C4 < 8 mg/dl), urine analysis, antinuclear antibodies (ANAs), anti-dsDNA, 24-h urine analysis for proteinuria, as classical diagnostic biomarkers for Lupus and LN.

### Sample size calculation

All eligible cases were collected along the study duration according to the inclusion criteria (convenient samples).

### Sample collection for analysis of lncRNAs expression

Two millilitres of blood were collected in EDTA-containing blood collection tubes from all subjects participating in this study and transferred immediately to the Medical Biochemistry and Molecular Biology Department, Mansoura Faculty of Medicine. Peripheral blood mononuclear cells (PBMCs) were isolated from blood samples using Ficoll-Hypaque density-gradient centrifugation and used for the separation of lncRNAs. Total RNA extraction was done using QIAzol Lysis Reagent in accordance with the manufacturer′s specifications (QIAGEN, Germany). The RNA concentration and purity were checked by Thermo Scientific NanoDrop One. Reverse transcription of 1ug of RNA was done using SensiFAST™ cDNA Synthesis Kit (Bioline, UK) on Applied Biosystems Proflex Thermal Cycler. cDNA templates were amplified using a real-time PCR instrument (Azure Cielo 6, Azure, USA) and primers specific for CTC-471J1.2 and *NeST* amplifications.

Quantitative real-time polymerase chain reaction (qRT-PCR) was done in 20 μl total reaction volume [10 μl of Bioline SYBR green PCR Master Mix (Bioline, UK), 1 μl of cDNA template, 1 μl (10 pmol/μl) for each forward and reverse gene primers, and 7 μl of nuclease-free water] using the following program: initial denaturation at 95 °C for 2 min followed by 40 cycles of 95 °C for 10 s and 60 °C for 30 s. β-actin was used as an endogenous reference gene to normalize the lncRNA expression levels.

The primer sets were designated using Primer3Plus software (http://www.bioinformatics.nl/cgi-bin/primer3plus/primer3plus.cgi), and primer specificity was checked using Primer-BLAST program (NCBI/ primer-BLAST (https://www.ncbi.nlm.nih.gov/tools/primer-blast/). Primers were purchased from Vivantis (Vivantis Technologies, Malaysia).

The sequences of the used primer pairs are supplied on Table 1. The specificity of each primer was confirmed by the presence of a single sharp peak by melting curve.

**Table 1:** The sequence of human primers used in qRT-PCR analysis

**Gene**	**Sequence**	**Product size**
**CTC-471J1.2**	**Forward primer:** ACAAATCTGAAAATACCACCTTG **Reverse primer:** TTTCCTAGAAATCATTTAACCCA	106 bp
***NeST***	**Forward:** AAGTTCTGGGCTTCTCCTCC Reverse: GACTTCAAAGAGTCTGAGGT	281 bp
**β-actin**	**Forward:** GTGGCCGAGGACTTTGATTG **Reverse:** GTGGGGTGGCTTTTAGGATG	104 bp

The relative expression levels of CTC-471J1.2 and *NeST* genes were calculated by ΔΔCt method, and the fold change of gene expression was expressed as 2^−ΔΔCT^ [18].

### Statistical analysis

Using IBM SPSS (Statistical package for social science) version 24 for Windows, data were coded, computed, and analyzed:

Several variables were recoded to improve the analysis’s strength:

- ❖ Descriptive statistics: were used to describe the demographic data, clinical and laboratory presentations of the cases
- ❖ Qualitative data were displayed using frequency tables (Number and percentages).
- ❖ For quantitative variables, the one-sample Kolmogorov-Smirnov test was used to determine the data’s normality before the data were displayed by central indices and dispersion:

Standard deviation (SD) and mean for variables with normally distributed data. Median and range (Minimum-Maximum) for variables with non-normal distribution.

- ➢ Analytical statistics:
  - ❖ Chi-square test and Fischer exact test were used for categorical variables, to compare between different groups as appropriate. To compare two groups under study, the student t test was applied to parametric quantitative variables while the Mann Whitney test was applied to non-parametric quantitative data.
  - ❖ Kruskal Wallis test; to compare between more than two studied groups when comparing non-parametric quantitative variables.
  - ❖ Sensitivity and specificity analysis were performed and Receiver operating characteristic (ROC) curve was plotted to assess the impact of LncRNAs (CTC-471J1.2, *NeST*) levels on disease activity
  - ❖ The analysis of binary logistic regression was performed to predict the independent factors of disease activity. Calculated odds ratios with a 95% confidence interval were adjusted odds ratios.
- ➢ Level of significance: For each statistical test, a result was deemed significant when the chance of error was 5% or less (*p* ≤ 0.05).

## Results

### The demographic, clinical, and laboratory data of JSLE participants

**Fig. 1:**
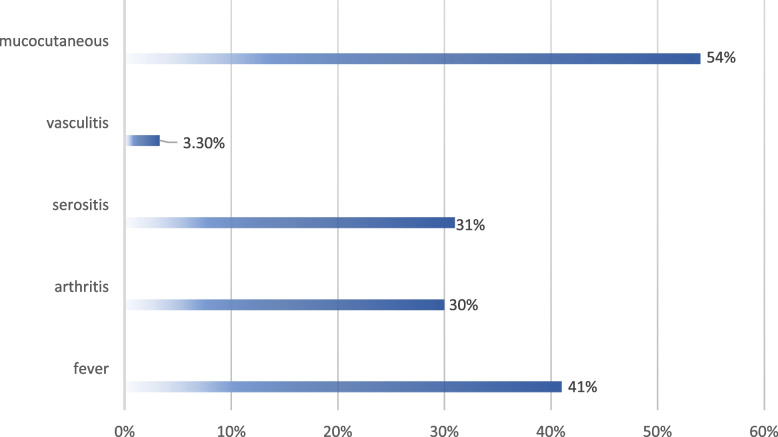
Clinical presentations of JSLE patients

Sixty-one JSLE patients enrolled in the study. Demographic, and clinical characteristics for patients are shown in Fig. 1 and Table 2. The majority of cases were males (75.4%). It is known males carry poor prognosis for kidney involvement as we strictly include SLE cases with LN. The mean age of cases was 13.8 years ± 2.56 SD, whereas the mean age of the disease onset was 11.3 ± 2.29 years, and the disease duration was between 0.1–7 years with a median of 2 years. The median SLEDAI was 0- 21 with a median of 4, while renal SLEDAI was 0–16 with a median of 2. The laboratory findings and medical therapy of the studied cases are summarized in Table 3. The mean of serum creatinine was 0.64 ± 0.21 SD, and that of complement was 98.4 ± 25.8 SD. Leucopenia was detected in 3 cases and thrombocytopenia in 2 cases. Patients were subdivided into two subgroups: 45 SLE patients with inactive nephritis and 16 SLE patients with active nephritis. Subsequently, we compared the patients’ subgroups in (Table 4), and our results revealed no statistically significant difference was found among the patients’ subgroups regarding the demographic, clinical, laboratory characteristics or the medical therapy (*P* > 0.05).

**Table 2:** Demographic, characteristic, damage index, and nephritis histopathology of juvenile systemic lupus erythematosus (JSLE) patients

Parameter	JSLE patients (*n* = 61)
Age /y: (mean ± SD)	13.8 ± 2.56
Sex (Male / Female): N (%)	46 (75.4)/15 (24.6)
BMI: n (%)	
Underweight (Less than the 5^th^ percentile)	1 (1.6)
Normal (5^th^ percentile to less than the 85^th^ percentile)	43 (70.5)
Overweight (85^th^ percentile to less than the 95^th^ percentile)	14 (23)
Obese (95^th^ percentile or greater)	3 (4.9)
Age of disease onset/y: (mean ± SD)	11.3 ± 2.29
Duration of disease/y: (median (range))	2 (0.1–7)
SLEDAI-2K: (median (range))	4 (0–21)
Disease activity status: n (%)	
High disease activity	21 (34.5)
Low disease activity	32 (52.5)
Inactive disease	8 (13)
SLICC/ACR-Damage Index: (median (range))	0 (0–0)
Nephritis: (mean ± SD)	3.03 ± 1.03
Class I: n (%)	5 (8.2)
Class II: n (%)	15 (24.6)
Class III: n (%)	15 (24.6)
Class IV: n (%)	25 (41)
Class V: n (%)	1 (1.6)
Active lupus nephritis: n (%)	
No	45 (73.8)
Yes	16 (26.2)
Activity index: (median (range))	6 (0–10)
Chronicity index: (median (range))	1 (0–4)
Renal SLEDAI: (median (range))	2 (0–16)

Values reported as median (range), percentile, and mean ± SD

*Abbreviations*: *n* Number, *y* Year, *JSLE* Juvenile systemic lupus erythematosus, *BMI* Bone mass index, *SLEDAI* Systemic Lupus Erythematosus Disease Activity Index, *SLICC/ACR damage index* Systemic Lupus International Collaborating Clinics/American Colleague of Rheumatology

**Table 3:** The laboratory findings and medical therapy in JSLE patients

Laboratory findings/Medical therapy	JSLE (*n* = 61)
24h urine protein (gm/dl): (median (range))	0.18 (0.1–5)
Serum creatinine (mg/dL): (mean ± SD)	0.64 ± 0.21
ESR (mm/hour): (mean ± SD)	36 ± 21.7
Complement (mg/dL): (mean ± SD)	98.4 ± 25.8
Anti-dsDNA (IU/mL): (mean ± SD)	548 ± 380.3
Anemia (< 10 g/dl): n (%)	
No	48 (78.7)
Yes	13 (21.3)
Leucopenia (< 5 × 10^3^/ µL): n (%)	
No	58 (95)
Yes	3 (5)
Thrombocytopenia (< 150 × 10 ^3^/µL): n (%)	
No	59 (96.7)
Yes	2 (3.3)
Elevated liver enzymes: n (%)	
No	56 (92)
Yes	5 (8)
APL antibodies: n (%)	
Negative	50 (82)
Positive	11 (18)
Medications: n (%)	
Steroid + Hydroxychloroquine	18 (29.5)
Steroid + Hydroxychloroquine + CYC	17 (27.9)
Steroid + Hydroxychloroquine + MMF	9 (14.8)
Steroid + Hydroxychloroquine + CYC + MMF	17 (27.9)

Values reported as median (range), percentile, and mean ± SD

*Abbreviations*: *n* Number, *SLE* Juvenile Systemic Lupus Erythematosus, *ESR* Erythrocyte Sedimentation Rate, *APL* Anti-phospholipid Antibodies, *CYC* Cyclophosphamide, *MMF* Mycophenolate Mofetil

**Table 4:** Comparison between active and inactive lupus nephritis (LN) regarding clinical presentations and laboratory findings

**Parameter**	**Inactive LN (*n* = 45)**	**Active LN (*n* = 16)**	**Test of Significance**
Sex			
Male	34 (75.6)	12 (75)	FET *P* = 1
Female	11 (24.4)	4 (25)	
Fever			
Absent	29 (64.4)	7 (43.8)	x^2^ = 2.1 *P* = 0.15
Present	16 (35.6)	9 (56.3)	
Mucocutaneous			
Absent	23 (51)	5 (31.2)	X^2^ = 1.87 *P* = 0.17
Present	22 (49)	11 (68.8)	
Arthritis			
Absent	32 (71)	11 (68.8)	FET *P* = 1
Present	13 (29)	5 (31.2)	
Serositis			
Absent	32 (71)	10 (62.5)	FET *P* = 0.54
Present	13 (29)	6 (37.5)	
Vasculitis			
Absent	45 (100)	14 (87.5)	FET *P* = 0.06
Present	0.0	2 (12.5)	
Medications			
2 medications	16 (35.6)	2 (12.5)	FET *P* = 0.11
> 2 medications	29 (64.4)	14 (87.5)	
Anemia			
Absent	33 (73.3)	15 (93.8)	FET *P* = 0.15
Present	12 (26.7)	1 (6.3)	
Leucopenia			
Absent	42 (93.3)	16 (100)	FET 0.56
Present	3 (6.7)	0.0	
Thrombocytopenia			
Absent	44 (97.8)	15 (93.8)	FET *P* = 0.45
Present	1 (2.2)	1 (6.3)	
Liver function test			
Normal	42 (93.3)	14 (87.5)	FET *P* = 0.6
Elevated	3 (6.7)	2 (12.5)	
APL antibodies			
Negative	36 (80)	14 (87.5)	FET *P* = 0.7
Positive	9 (20)	2 (12.5)	

Values reported as percentile

*P* > 0.05 is considered statistically not significant

*Abbreviations*: *n* Number, *LN* Lupus nephritis, *APL* Anti-phospholipids antibodies, *FET* Fischer exact test, *X*^*2*^ Chi square test

### Expression levels of LncRNAs (CTC-471J1.2, *NeST*) in the studied subjects

**Fig. 2:**
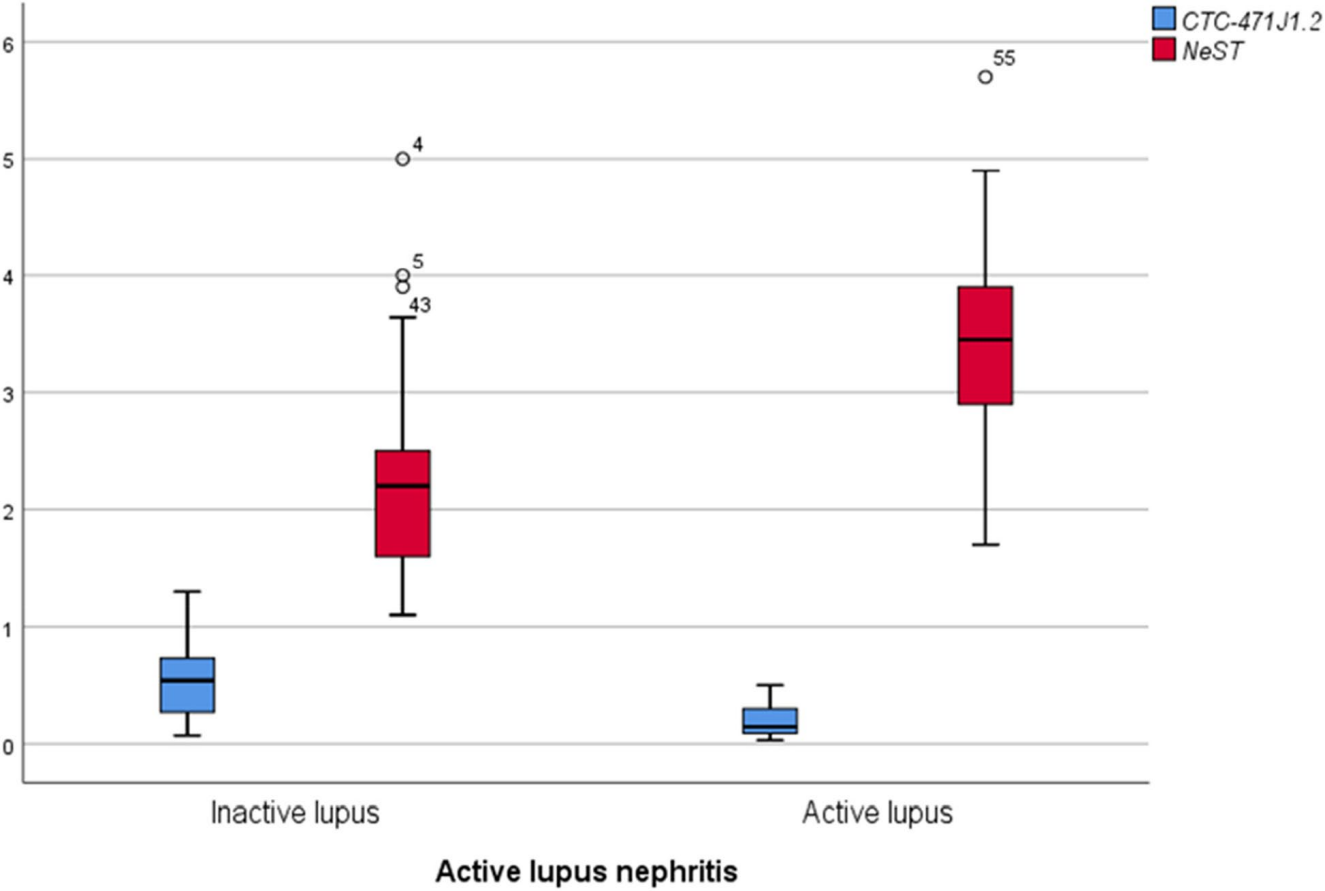
Box plots showing the summaries of CTC-471J1.2 and *NeST* findings among inactive and active lupus nephritis

Expression profiling of lncRNAs-CTC-471J1.2 in PBMCs from inactive and active lupus nephritis patients showed a significant decrease as compared to controls (*P*_1_ = 0.02, *P*_2_ < 0.001, respectively). In contrast, the expression pattern of *NeST* was significantly increased in patients with inactive or active lupus nephritis (P_1_, *P*_2_ < 0.001) as compared to controls. Furthermore, the expression level of CTC-471J1.2 was significantly increased (*P* < 0.001) in patients with inactive lupus nephritis than in those with active lupus nephritis. The patients with inactive LN also displayed a significantly lower (*P* < 0.001) level of *NeST* expression than the active LN group (Table 5, Fig. 2).

**Table 5:** Comparison of the expression level of CTC-471J1.2 and *NeST* among inactive, active lupus nephritis patients, and controls

**Parameter**	**Inactive LN (*n* = 45)**	**Active LN (*n* = 16)**	**Controls (*n* = 40)**	**Test of significant**
CTC-471J1.2	0.54 (0.07–1.3)	0.14 (0.03–0.5)	0.9 (0.25–1.1) *P*_1_ = 0.02* *P*_2_ < 0.001*	KW *P* < 0.001*
*NeST*	2.2 (1.1–5)	3.4 (1.7–5.7)	1.1(0.3–1.8) *P*_1_ < 0.001* *P*_2_ < 0.001*	KW *P* < 0.001*

Values reported as median (min-max)

*Abbreviations*: *n* Number, *LN* Lupus nephritis, *KW* Kruskal-Wallis test used to compare non-parametric variables, *P*_*1*_ Significant difference between inactive lupus and controls, *P*_*2*_ Significant difference between active lupus and controls, *P* Significant difference between inactive and active lupus

*P, *P_1_, *P_2_ < 0.05 are considered statistically significant

### Correlation between LncRNAs (CTC-471J1.2, *NeST*) expression levels and characteristics of lupus disease activity parameters

Detailed correlation analysis of CTC-471J1.2 and *NeST* levels with various clinical and laboratory parameters of the studied subjects are listed in Table 6. LncRNA-CTC-471J1.2 were negatively correlated with renal SLEDAI (*r* = -0.3, *P* = 0.04), 24-h urine protein (*r* = -0.3, *P* = 0.013), Anti-dsDNA titer (*r* = -0.27, *P* = 0.048) and *NeST* expression level (*r* = -0.46, *P* < 0.001). In contrast, the expression level of CTC-471J1.2 had a significantly positive correlation with complement (*r* = 0.28, *P* = 0.007). Regarding the expression level of *NeST*, there was no significant correlation with the SLE clinical or laboratory features (*P* > 0.05).

**Table 6:** Correlation between the expression level of CTC-471J1.2 and *NeST* and characteristics of SLE disease activity

Parameter	CTC-471J1.2	*NeST*
*r* (*P*- value)		
Renal SLEDAI	-0.3 (0.04)*	-0.2 (0.2)
SLEDAI-2K	-0.05 (0.7)	-0.16 (0.3)
Disease activity status	0.06 (0.9)	0.26 (0.09)
24 h urine protein(gm/dl)	-0.3 (0.013)*	0.12 (0.37)
Complement	0.28 (0.007)*	-0.36 (0.09)
Anti-dsDNA	-0.27 (0.048)*	0.08 (0.54)
*NeST*	-0.46 (< 0.001)*	-

*Abbreviations*: *SLE* Systemic lupus erythematosus, *SLEDAI* Systemic Lupus Erythematosus Disease Activity Index, *Anti-ds-DNA* Anti-double-stranded DNA, *r* Spearman correlation coefficient

*P < 0.05 is considered statistically significant

### ROC curve analysis

**Fig. 3:**
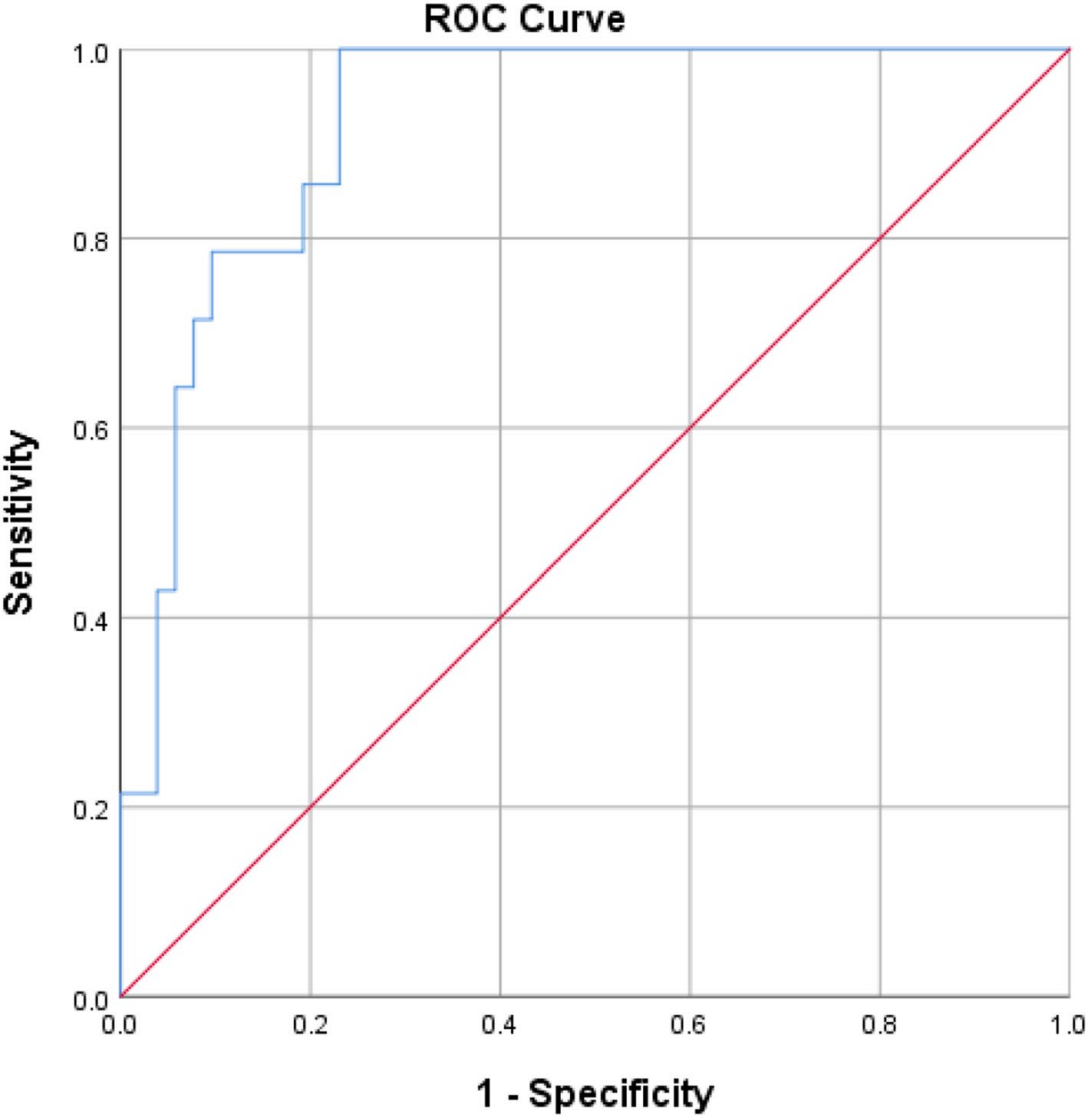
ROC curve of use of combined CTC-471J1.2 and *NeST* levels to discriminate disease activity

To investigate the diagnostic utility of lncRNAs-CTC-471J1.2 and *NeST* in differentiation of active LN cases, we found the following: CTC-471J1.2 at a cut-off value of 0.25 provided a sensitivity of 85% and a specificity of 83% with an area under curve (AUC) of 0.84. *NeST* sensitivity was 80% and specificity of 71% at the cut-off point of 2.75 with AUC of 0.83. It was found that the combined utilization of lncRNAs-CTC-471J1.2 and *NeST* had sensitivity of 93%, specificity of 77% with AUC of 0.92 (Table 7, Fig. 3).

**Table 7:** Sensitivity analysis/ROC curve of CTC-471J1.2, *NeST* expression levels to discriminate disease activity

**Parameter**	**AUC**	**Cut off**	**Sensitivity**	**Specificity**	**95% CI**	***P*-value**
CTC-471J1.2	0.84	0.25	85%	83%	(0.72–0.94)	< 0.001*
*NeST*	0.83	2.75	80%	71%	(0.725–0.966)	< 0.001*
Combined: (CTC-471J1.2, NeST)^a^	0.92	-	93%	77%	(0.85–0.98)	< 0.001*

*AUC* Area Under Curve, *CI* Confidence Internal

*P < 0.05 is considered statistically significant

^a^Assessed by saved probabilities of logistic regression

### Logistic regression analysis of lncRNAs (CTC-471J1.2, *NeST*) levels for prediction of active lupus nephritis

This analysis revealed that CTC-471J1.2 and *NeST* were found as independent predictors of disease activity (Table 8).

**Table 8:** Logistic regression analysis of CTC-471J1.2, *NeST* expression levels with the presence of active lupus nephritis

	**Regression coefficient**	***P* value**	**Odds ratio (OR)**	**95% CI**
CTC-471J1.2	-5.74	0.012*	3.18	(1.24–7.8)
*NeST*	1.16	0.02*	0.003	(0–0.39)
Constant	-1.07			
Predicted%	83.6%			
Model χ2	12.1, *P* = 0.001*			

*CI* Confidence Internal

*P < 0.05 is considered statistically significant

## Discussion

JSLE is a multisystem autoimmune disease with inflammatory consequences. It shows marked heterogeneity between patients, causing manifestations ranging from mild to severe [19]. LN is one of the most severe manifestations of SLE associated with considerable morbidity and mortality [2]. Dysregulation of lncRNAs function has been identified in cancer as well as autoimmune diseases via different mechanisms to alter encoding gene expression [20, 21]. There is mounting evidence that the lncRNAs’ expression plays a significant role in the pathogenesis of SLE and lupus nephritis by acting as a regulator of immune and inflammatory response [22]. It can also be used as a tool for evaluating renal outcome of LN patients [23].

In the current study, we aimed to investigate the expression of lncRNA-CTC-471J1.2 and *NeST* in pediatric lupus nephritis patients. Regarding CTC-471J1.2 expression, it was significantly downregulated in LN cases compared to controls, with higher level of expression among inactive LN patients than active LN group. Likewise, findings of Luo et al., 2018 who proposed that the downregulation in the expression level of lncRNAs including CTC-471J1.2 was associated with biological processes, cellular components, and molecular function, which affects several gene pathways, such as cytokine-cytokine receptor interaction, TNF signalling pathway, MAPK signalling pathway, and NF-κB signalling pathways [24].

Saleh et al., 2019 reported in agreement with our results that the cell-free lncRNA-CTC-471J1.2 was considered a potential biomarker for the diagnosis of SLE. It was revealed to be the most specific and sensitive diagnostic biomarker among the studied markers for lupus nephritis [9, 24]. By utilizing the ROC curve, CTC-471J1.2 appears to be a potential diagnostic biomarker for lupus nephritis activity, with high sensitivity (85%) and specificity (83%).

Moreover, there was a significant negative correlation between CTC-471J1.2 and SLE activity parameters, specifically renal SLEDAI, 24 h urine protein, and anti-dsDNA titres. This is in accordance to the results of Mihaylova et al., 2020 who found that CTC-471J1.2 expression levels has a negative correlation with SLEDAI scores in all SLE patients and a positive correlation with eGFR in only LN patients [25]. As the decrement of complement is a sign of disease activity [24], CTC-471J1.2 profile was positively correlated with its level. Our study reported a significant relationship between CTC-471J1.2 and *NeST* lncRNAs.

*NeST* is a long intergenic non-coding RNAs (lincRNAs) that function through transcriptional regulation. It is located near the IFN-γ-encoding gene in both mouse and human, and it can upregulate the expression of the IFN‑γ gene pathway [10]. It is known that IFN-γ plays a principal role in the development of proliferative LN [26] and it was significantly elevated in cases with active LN [27]. Hence, *NeST* was hypothesized to be involved in the pathogenesis of proliferative LN by regulating inflammatory chemokines and T-helper cells.

Compared with matched controls, *NeST* expression was upregulated among JSLE cases, with higher levels among our active LN cohort. This finding is running with the preceding report from Li et al. [28] and Xiao et al. [29]. Its level of expression was found to be upregulated in several immune diseases such as Sjögren syndrome and rheumatoid arthritis [30] as well.

However, *NeST* did not show any correlation with the various nephritis activity parameters. Nevertheless, the results of correlation should be considered with caution owing to the limited sample size. Further, on applying ROC curve for *NeST,* it displayed a lower sensitivity (80%) and specificity (71%) than CTC-471J1.2. Thus, concluding that lncRNA-CTC-471J1.2 seems to be a better epigenetic biomarker for LN activity as asserted by previous studies [9, 25].

On applying ROC curve for CTC-471J1.2 and *NeST* expression levels as biomarkers for disease activity, the sensitivity raised to 93% and we found that the panel of both significantly increased the AUC value to 0.92 compared with when they were utilized individually.

Regarding the potential for the development of active lupus nephritis, our study for the first time reported that CTC-471J1.2 and *NeST* work as significant predictors of active LN. Additionally, CTC-471J1.2 was shown to be a better predictor of disease activity than *NeST* as inferred from this prediction model.

There are some limitations of our study, the sample size was relatively small, we did not correlate between lncRNAs and the histological findings of LN nor did correlate them with other conditions with renal involvement, as post-streptococcal glomerulonephritis. Therefore, large-scale studies in different populations are pivotal to confirm our findings.

## Conclusion

We found that the lncRNAs (CTC-471J1.2 and *NeST*) were preferentially expressed in LN. CTC-471J1.2 was significantly correlated with disease activity parameters, and it appears the most specific and sensitive diagnostic biomarker for nephritis. Furthermore, both CTC-471J1.2 and *NeST* could serve as predictors for lupus nephritis activity.

## Data Availability

All data generated during this study are in this published article.

## Abbreviations

ANAs: Antinuclear antibodies
AUC: Area under curve
CBC: Complete blood count
CRP: C-reactive protein
eGFR: Estimated glomerular filtration rate
ESR: Erythrocyte sedimentation rate,
EULAR/ACR: European League Against Rheumatism/American College of Rheumatology
ISN: International Society of Nephrology
JSLE: Juvenile systemic lupus erythematosus
LincRNAs: Long intergenic non-coding RNAs
LN: Lupus nephritis
lncRNAs: Long-non-coding RNAs
MUCH: Mansoura University Children’s Hospital
*NeST*: Nettoie Salmonella pas Theiler’s
NIH: National Institutes of Health
PBMCs: Peripheral blood mononuclear cells
qRT-PCR: Quantitative real-time polymerase chain reaction
ROC: Receiver operating characteristic
RPS: Renal Pathology Society
SD: Standard deviation
SLEDAI-2K: SLE Disease Activity Index 2000
SLICC/ACR: Systemic Lupus International Collaborating Clinics/ACR
SPSS: Statistical package for social science
Tmevpg1: Theiler’s murine encephalomyelitis virus persistence candidate gene 1
